# Prognostic relevance of longitudinal HGF levels in serum of patients with ovarian cancer

**DOI:** 10.1002/1878-0261.12949

**Published:** 2021-04-02

**Authors:** Daniel Martin Klotz, Theresa Link, Pauline Wimberger, Jan Dominik Kuhlmann

**Affiliations:** ^1^ Department of Gynecology and Obstetrics Medical Faculty and University Hospital Carl Gustav Carus Technische Universität Dresden Germany; ^2^ National Center for Tumour Diseases (NCT) Dresden Germany; ^3^ German Cancer Research Center (DKFZ) Heidelberg Germany; ^4^ Faculty of Medicine University Hospital Carl Gustav Carus Technische Universität Dresden Germany; ^5^ Helmholtz‐Zentrum Dresden‐Rossendorf (HZDR) Germany; ^6^ Dresden and German Cancer Research Center (DKFZ) German Cancer Consortium (DKTK) Heidelberg Germany

**Keywords:** biomarker, cMET, HGF, ovarian cancer, prognosis

## Abstract

The pleiotropic protein hepatocyte growth factor (HGF) is the only known ligand of the tyrosine kinase mesenchymal–epithelial transition (cMET) receptor. The HGF/cMET pathway mediates invasion and migration of ovarian cancer cells, and upregulation of HGF/cMET pathway components has been associated with poor prognosis. This study investigated the clinical relevance of circulating HGF in serum of patients with ovarian cancer. Serum HGF (sHGF) was determined by enzyme‐linked immunosorbent assay in a total of 471 serum samples from 82 healthy controls and 113 patients with ovarian cancer (88.5% with ≥ FIGO III). Patient samples were collected at primary diagnosis and at four follow‐up time points throughout treatment and at disease recurrence. Patients with ovarian cancer showed elevated median sHGF levels at primary diagnosis, and sHGF levels transiently increased after surgery and normalized in the course of chemotherapy, even dropping below initial baseline. Higher levels of sHGF were an independent predictor for shorter overall survival (OS) (a) at primary diagnosis (HR = 0.41, 95% CI: 0.22–0.78, *P* = 0.006), (b) at longitudinal follow‐up time points (after surgery and before/during/after chemotherapy), (c) along the patients’ individual dynamics (HR = 0.21, 95% CI: 0.07–0.63, *P* = 0.005), and (d) among a subgroup analysis of patients with BRCA1/2 wild‐type ovarian cancer. This is the first study proposing sHGF as an independent prognostic biomarker for ovarian cancer at primary diagnosis and in the course of platinum‐based chemotherapy, irrespective of the postoperative residual disease after surgical debulking. sHGF could be implemented into clinical diagnostics as a CA125 auxiliary tumor marker for individualized prognosis stratification and sHGF‐guided therapy monitoring.

## Graphical abstract

The HGF/cMET signaling axis contributes to tumorigenesis in various cancers. Here, we describe that sHGF can be used as an independent biomarker in ovarian cancer for prognostic stratification at primary diagnosis and in the course of first‐line platinum‐based chemotherapy. The biomarker sHGF is an easily detectable serum parameter and increased in patients with ovarian cancer compared with healthy individuals. It could possibly be implemented into routine clinical practice for personalised prognosis stratification (high and low risk) in ovarian cancer and for monitoring treatment response.

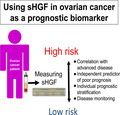

**Abbreviations**
HGF: hepatocyte growth factor
cMET: mesenchymal–epithelial transition
sHGF: serum HGF
OS: overall survival
PFS: progression‐free survival
HR: hazard ratio
FIGO: Fédération Internationale de Gynécologie et d'Obstétrique
CI: confidence interval
ROC: receiver operating characteristic
OR: odds ratio
AUC: area under the curve
ED: estimated difference
BMI: body mass index
wtBRCA1/2: BRCA1/2 wild‐type
CTC: circulating tumor cell

## 1 Introduction

Ovarian cancer is the leading cause of death from gynecological malignancies, and more than 70% of patients are diagnosed with advanced disease [1]. Standard therapy of advanced ovarian cancer includes surgical debulking, aiming at macroscopic complete tumor resection and platinum/paclitaxel‐based chemotherapy and maintenance treatment with bevacizumab [2, 3, 4]. High‐grade serous histology, advanced disease, and platinum resistance are associated with poor prognosis. The most important clinical prognostic factor in advanced ovarian cancer is the postoperative residual tumor burden [1, 5]. Despite improved radical surgical debulking and the recent implementation of novel targeted therapies with PARP inhibitors as maintenance treatment after standard first‐line therapy, the majority of patients with advanced ovarian cancers still face a poor overall prognosis [6, 7, 8, 9]. Therefore, the discovery of blood‐based predictive and/or prognostic biomarkers for ovarian cancer patients is of high clinical priority.

The pleiotropic protein hepatocyte growth factor (HGF) was originally discovered in 1984 as a mitogen for primary hepatocytes [10]. So far, HGF is the only known endogenous ligand of the receptor tyrosine kinase mesenchymal–epithelial transition (c*MET*), which is encoded by the *MET* proto‐oncogene [11, 12]. The canonical activation of the HGF/cMET signaling axis is initiated by the binding of HGF to cMET on the cellular surface, inducing cMET homodimerization and autophosphorylation, followed by the activation of key downstream pathways, such as the phosphoinositide‐3‐kinase pathway [13, 14]. HGF signaling plays a role in the development of normal ovaries and follicles through paracrine signaling between HGF‐ and cMET‐expressing cells [15, 16].

The HGF/cMET pathway controls diverse cellular functions, such as proliferation, angiogenesis, and migration, and has been associated with the metastatic progression of several human cancers [17, 18]. In ovarian cancer, the HGF/cMET pathway is aberrantly activated. Histologically detected overexpression of cMET (between 10% and 75%) has been observed across all histologic subtypes [19] and was associated with poor prognosis [20, 21]. There is also evidence from *in vitro* studies that HGF signaling contributes to matrix metalloproteinase 9‐mediated invasion and migration of ovarian cancer cells [22]. Consequently, HGF/cMET signaling has been exploited as a therapeutic target in clinical trials on ovarian cancer [23, 24]. Furthermore, it was proposed that circulating serum HGF (sHGF) levels are significantly raised in ovarian cancer patients at primary diagnosis compared with patients with borderline tumors or benign disease, and that elevated sHGF levels indicate poor prognosis [25]. Despite these preliminary findings, the clinical relevance of sHGF for ovarian cancer patients, especially among longitudinal serum samples in the course of primary treatment and disease relapse, is unknown.

Therefore, the objective of the present study was to investigate whether sHGF levels at (a) primary diagnosis or (b) among longitudinal follow‐up samples in the course of treatment (debulking surgery and platinum‐based chemotherapy) carry predictive and/or prognostic implications as a blood‐based biomarker for ovarian cancer.

## 2 Materials and methods

### 2.1 Patient characteristics

Patients were recruited, and samples were obtained and processed at the Department of Gynecology and Obstetrics at the Carl Gustav Carus University of Dresden, Technische Universität Dresden, Germany. Overall, 113 patients with histologically confirmed primary epithelial ovarian cancer (primary diagnosis from 2004 to 2019), who received cytoreductive surgery with the aim of macroscopic complete resection and the recommendation of platinum‐ and paclitaxel‐based chemotherapy in line with national guidelines, were included. OS was calculated from primary diagnosis and progression‐free survival (PFS) from the last administration of chemotherapy, and patients were clinically classified as primary platinum‐resistant if they recurred within 6 months after the completion of platinum‐based chemotherapy. All study participants gave written informed consent, and the Local Research Ethics Committee in Dresden approved the study (EK74032013) [26], and the study methodologies conformed to the standards set by the Declaration of Helsinki. The patients' clinical data are summarized in Table 1. Ovarian cancer was reported in agreement with the WHO classification of tumors derived from female genital tract, and staging was documented according to the Fédération Internationale de Gynécologie et d'Obstétrique (FIGO) [27], revised in 2014 [28]. FIGO stage was reported according to the revised version for all patients who underwent primary surgery from 2014 to 2019. In the case of no contraindications, patients with a tumor stage of at least FIGO IIIb were additionally treated with the monoclonal antibody bevacizumab.

**Table 1:** Patient characteristics.

*N* (total patient cohort)	113
Age	Median 60 years (35–82 years)
BMI	
Recorded	101 (89.4%) median 25.9 (17.1–50.3)
Unknown^a^	12 (10.6%)
FIGO	
I–II	13 (11.5%)
III–IV	100 (88.5%)
Histologic type	
Serous	101 (89.4%)
Other	12 (10.6%)
Residual tumor	
Macroscopic complete resection	51 (45.1%)
Any residual tumor	62 (54.9%)
BRCA status	
Unknown	56 (49.6%)
BRCA1/2 mutation	23 (20.4%)
No BRCA1/2 mutation	34 (30.0%)
Recurrence	
PFS	Median 13 months (1–140 months)
No relapse	35 (31.0%)
Relapse	78 (69.0%)
Survival	
OS	Median 35 months (1–188 months)
Alive	61 (54.0%)
Dead	52 (46.0%)

^a^ Cases where samples were obtained only at disease recurrence and surgery performed > 10 years ago, and no recording was made available.

### 2.2 Healthy controls

Eighty‐two female healthy individuals without a past medical history of benign disease or malignancy were recruited. Informed written consent was obtained from all donors, and the Local Research Ethics Committee in Dresden approved the study (EK74032013). Control sample acquisition was performed, according to the Declaration of Helsinki. In order to allow comparison, preparations of control samples and patient samples were performed with the same protocol [26].

### 2.3 Serum preparation

Serum preparation was performed as previously described [26]. After blood withdrawal with a 7.5 mL S‐Monovette® (Sarstedt AG & Co., Nuembrecht, Germany), blood samples were incubated at room temperature for at least 30 min to allow complete blood coagulation. Within 1 h after blood drawing, serum was prepared by centrifugation for 8 min at 1800 ***g*** at room temperature. The obtained cell‐free serum fraction was immediately frozen at −80 °C until further processing. Unnecessary freeze–thaw cycles were avoided. For analysis, samples were thawed on ice and were immediately processed after complete thawing. Samples were blinded so that neither time of blood drawing nor any other information could be disclosed during investigation.

### 2.4 Detection of sHGF

Serum HGF concentrations were determined with the commercially available enzyme‐linked immunosorbent assay kit HGF ELISA kit (Thermo Fisher, Waltham, USA), and the assays were performed, according to the manufacturer’s instructions. The optical readout was conducted with the microplate reader Infinite M200 and software magellan version 7.2 (Tecan, Männedorf, Switzerland).

### 2.5 Statistical analysis

The statistical analysis was conducted with R, version 3.6.2, and graphpad prism version 8.4.3 (GraphPad Software, La Jolla, CA, USA), adapted from Ref. [26]. In short, *P*‐values < 0.05 were considered statistically significant. All confidence intervals (CIs) were specified as 95% CI. Nonparametric, two‐tailed Mann–Whitney *U*‐test was used to compare sHGF levels. Using receiver operating characteristic (ROC) curve analysis, we assessed the capacity of sHGF concentrations to discriminate between ovarian cancer patients and healthy controls. The Hodges–Lehman estimate was used to determine the estimated differences (ED) of medians. Uni‐ and multivariate Cox proportional hazard regression model analyses were performed to study the prognostic relevance of sHGF, and hazard ratios (HRs) are indicated with 95% CI. To predict platinum resistance, univariate and multivariate generalized linear model analyses were performed and odds ratio (OR) are indicated with 95% CI. The Kaplan–Meier analyses were performed with significance levels indicated by log‐rank (Mantel–Cox) analysis, and HRs (Mantel–Haenszel) are shown with 95% CI. The correlation between sHGF levels at different time points or with age and CA125 was assessed by nonparametric Spearman’s correlation. For all relevant analyses, the cutoff for the ‘sHGF‐high’ or the ‘sHGF‐low’ subgroups was defined using maximally selected rank statistics, using conditional Monte Carlo for *P*‐value approximations (Fig. S1).

## 3 Results

### 3.1 Median sHGF level is highly elevated at primary diagnosis of ovarian cancer and is normalized by chemotherapy

**Fig. 1:**
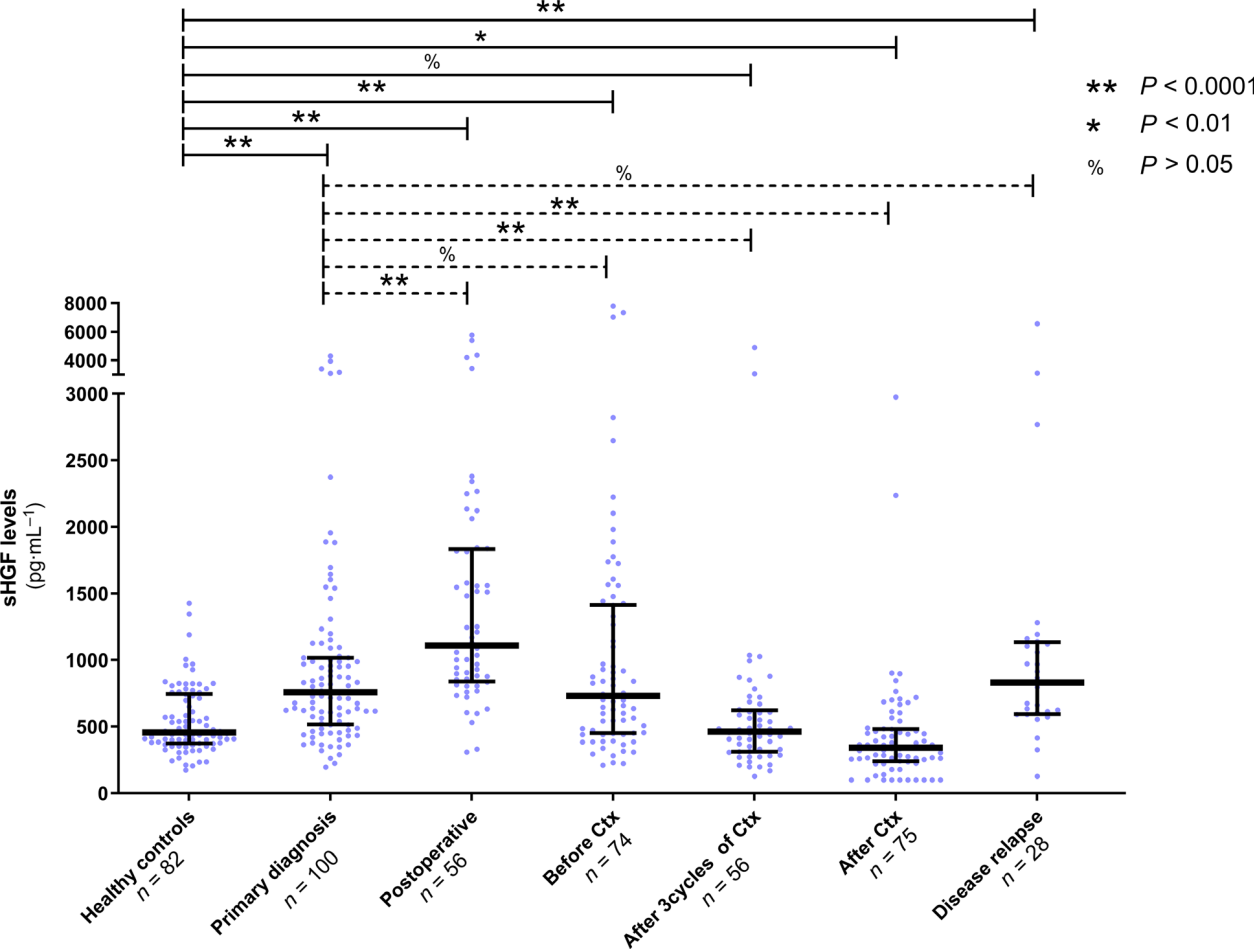
sHGF levels from primary diagnosis, surgery, and platinum‐based chemotherapy to relapse. Scatter plot showing sHGF levels in healthy controls (*n* = 82), in ovarian cancer patients at primary diagnosis (*n* = 100) and among five subsequent follow‐up samples, obtained (a) within 1 week after primary surgery (postoperative, *n* = 56), (b) before platinum‐based chemotherapy (before Ctx, *n* = 74), (c) after the third cycle of chemotherapy (*n* = 56), (d) after completion of chemotherapy (after Ctx, *n* = 75), and (e) at disease relapse (*n* = 28). The black horizontal lines indicate the median sHGF level in each group with error bars, showing the interquartile range. *P*‐values, according to the nonparametric, two‐tailed Mann–Whitney *U*‐test, are indicated.

**Fig. 2:**
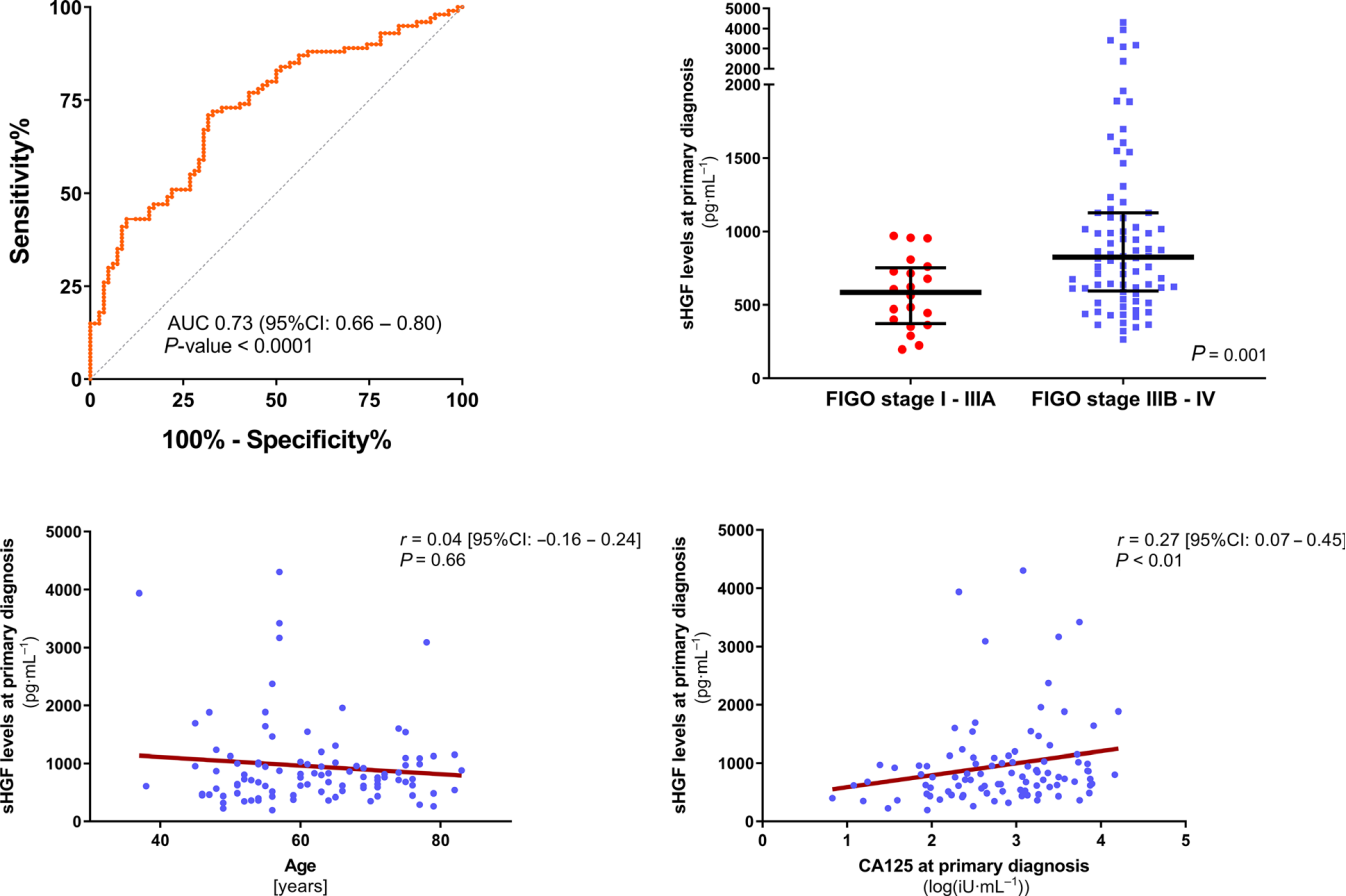
Association of sHGF level with clinicopathological parameters of ovarian cancer. (A) ROC analysis to determine the diagnostic ability of sHGF level to distinguish between patients with a primary diagnosis of ovarian cancer and healthy controls. Ovarian cancer patients, *n* = 100; healthy individuals, *n* = 82. The respective AUC value and the 95%CI are indicated. (B) Scatter plots comparing sHGF level at primary diagnosis between FIGOI‐IIIA (*n* = 20) vs. FIGO IIIB‐IV (*n* = 80) ovarian cancer patients. The black horizontal lines indicate the median sHGF level with error bars, showing the interquartile range. *P*‐value, according to the nonparametric, two‐tailed Mann–Whitney *U*‐test, is indicated. (C, D) Spearman’s correlation analysis of sHGF at primary diagnosis and (C) the patients’ age, *n* = 100 ovarian cancer patients; or (D) baseline CA125 log values, *n* = 97 ovarian cancer patients with available matching CA125 values at primary diagnosis.

We quantified sHGF levels in a comprehensive cohort of clinically documented ovarian cancer patients (*n* = 113) and compared it with levels in healthy controls (*n* = 82). The median sHGF level at primary diagnosis was significantly elevated compared with healthy controls with an ED of 241.8 ng·mL^−1^ (95% CI: 157.3–337.3 ng·mL^−1^, *P* < 0.0001; Fig. 1). Similarly, measuring sHGF levels showed a reasonable discriminative power between patients and controls with an area under the curve (AUC) of 0.73 (95% CI: 0.66–0.80, *P* < 0.0001) in the total patient cohort (Fig. 2A). When considering patients with FIGOI‐II ovarian cancer only, the AUC was 0.50 (95% CI: 0.31–0.69, *P* = 0.97; Fig. S2A).

We determined sHGF levels at primary diagnosis, within 1 week after primary surgery and at additional follow‐up time points in the course of chemotherapy. Samples at primary diagnosis were collected at hospital admission before cytoreductive surgery. The four subsequent serum samples were as follows: (a) within 1 week after debulking surgery (*n* = 56), (b) before initiating platinum‐based chemotherapy (*n* = 74), (c) after the first three cycles of chemotherapy (*n* = 56), and (d) after the completion of chemotherapy (*n* = 75; Fig. 1). After primary surgery, there was a strong yet transient increase in the median sHGF level compared with the baseline level at primary diagnosis with an ED of 393.4 ng·mL^−1^ (95%CI: 235.4–571.2 ng·mL^−1^, *P* < 0.0001). This then dropped again at the onset of platinum‐based chemotherapy to a level similar to primary diagnosis. Already after the first three cycles of platinum‐based chemotherapy, the median sHGF level was lower than the initial level at primary diagnosis (ED of −276.5 ng·mL^−1^, 95%CI: −388.4 to −171.7 ng·mL^−1^, *P* < 0.0001), ultimately dropping below the median level of healthy controls after chemotherapy with an ED of −90.2 ng·mL^−1^ (95%CI: −147.9.8 to to 30.9 ng·mL^−1^, *P* = 0.005). The median sHGF level rose again at disease recurrence, paralleling that of primary diagnosis (Fig. 1). Since median sHGF levels seemed to reflect a potential response to primary treatment, we further examined whether sHGF levels were associated with known clinicopathological characteristics.

### 3.2 Association of sHGF level with clinicopathological characteristics and platinum sensitivity of ovarian cancer patients

A higher level of sHGF at primary diagnosis was associated with advanced disease, that is, FIGO stage IIIB or higher (ED = 260.6 ng·mL^−1^, 95% CI: 101.8–448.3 ng·mL^−1^, *P* = 0.001; Fig. 2B), but it was unrelated to patients’ age (*r* = 0.04, 95% CI: −0.16 to 0.24, *P* = 0.66; Fig. 2C) or to the underlying histologic subtype (ED between nonserous vs. serous = −152.6 ng·mL^−1^, 95% CI: −1530 to 130.0 ng·mL^−1^, *P* = 0.29; Fig. S2B). There was no association between sHGF and the postoperative residual tumor, neither at the time of primary diagnosis nor after debulking surgery (Fig. S3). A weak correlation between sHGF at primary diagnosis and serum CA125 was observed (*r* = 0.27, 95% CI: 0.07–0.45, *P* = 0.008; Fig. 2D). Despite similar median values (Fig. 1), there was no correlation between sHGF levels at primary diagnosis and at disease recurrence, as determined from 15 available matched serum samples at these time points. Moreover, sHGF was prognostically noninformative among all 28 available serum samples at disease recurrence (Fig. S4).

In our patient cohort, 12 of 113 patients (10.6%) were retrospectively identified as primary platinum‐resistant. There was no significant difference between the median sHGF level in platinum‐resistant in platinum‐sensitive patients at primary diagnosis (844.5 vs. 713.3 ng·mL^−1^, *P* = 0.08). Likewise, sHGF level was not predictive for platinum resistance at any time point (Fig. S5).

### 3.3 sHGF at primary diagnosis is an independent predictor of survival

**Fig. 3:**
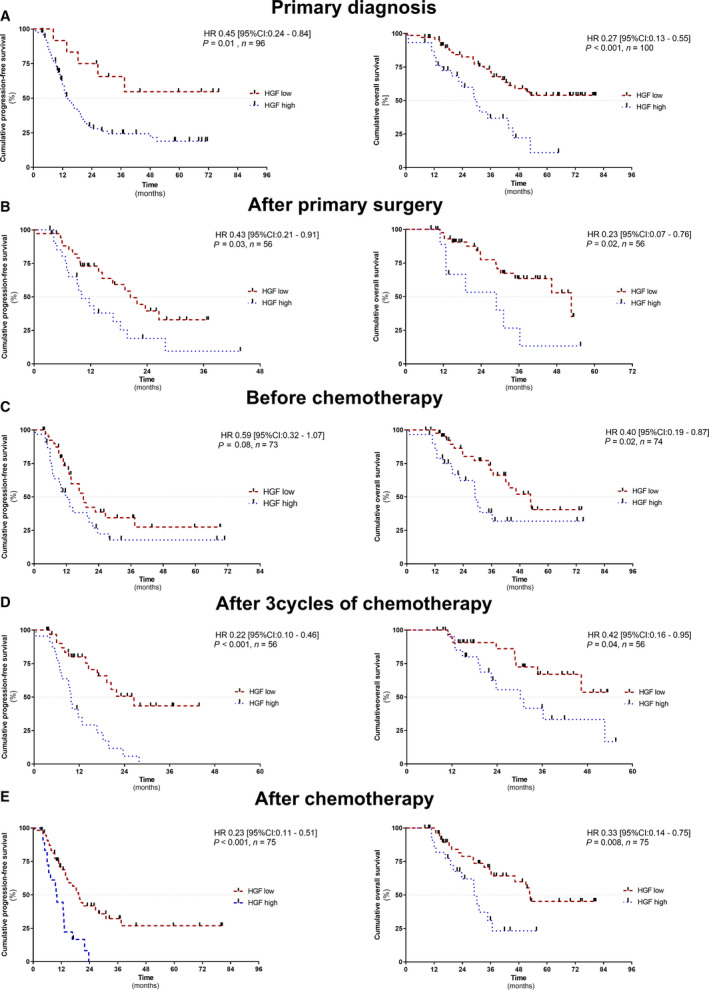
Prognostic relevance of sHGF at primary diagnosis, surgery, and throughout platinum‐based chemotherapy. The Kaplan–Meier analyses comparing progression‐free survival (PFS) and OS of patients with high sHGF level vs. patients with low sHGF level (A) at primary diagnosis, *n*_(PFS)_ = 96 and *n*_(OS)_ = 100; (B) after primary surgery, *n*_(OS and PFS)_ = 56; (C) before initiating chemotherapy, *n*_(PFS)_ = 73 and *n*_(OS)_ = 74; (D) after three cycles of chemotherapy, *n*_(PFS and OS)_ = 56; and (E) after chemotherapy, *n*_(PFS and OS)_ = 75. *P*‐values, HR, and 95%CI were calculated, as described in Materials and methods section.

Univariate Cox proportional hazard regression analysis (univariate analysis) revealed that a higher sHGF level at primary diagnosis indicated reduced PFS (HR = 0.32, 95% CI: 0.13–0.81, *P* = 0.016) and reduced OS (HR = 0.35, 95% CI: 0.19–0.64, *P* < 0.001; Fig. S6). The Kaplan–Meier survival analysis was also performed. Consistently, patients with a high sHGF level had shorter PFS (HR = 0.45, 95% CI: 0.24–0.84, *P* = 0.01) and shorter OS (HR = 0.27, 95% CI: 0.13–0.55, *P* < 0.001; Fig. 3A).

Next, we performed multivariate Cox proportional hazard regression analysis (multivariate analysis) with PFS and OS as outcome variables. We included sHGF levels and known risk factors of ovarian cancer, that is, age, body mass index (BMI), postoperative residual tumor, and FIGO stage. We observed that an elevated sHGF level at primary diagnosis was an independent predictor of shorter OS (HR = 0.41, 95% CI: 0.22–0.78, *P* = 0.006) but not PFS (HR = 0.42, 95% CI: 0.16–1.07, *P* = 0.069; Fig. S7).

### 3.4 sHGF levels at single time points throughout primary treatment are independent predictors of survival

The prognostic information of sHGF at primary diagnosis is important to identify patients at high risk of relapse before the start of therapeutic interventions. In order to analyze whether sHGF could also be used for therapy monitoring, we additionally performed independent survival analyses at all longitudinal follow‐up time points (Fig. 1). According to univariate analysis, a high level of sHGF at all investigated time points, with the exception of the time point before the initiation of platinum‐based chemotherapy, indicated shorter PFS (after surgery: HR = 0.45, 95% CI: 0.23–0.90, *P* = 0.024; after the first three cycles of chemotherapy: HR = 0.26, 95% CI: 0.13–0.54, *P* < 0.001; and after the completion of chemotherapy: HR = 0.35, 95% CI: 0.19–0.64, *P* < 0.001; Fig. S6). Moreover, a high level of sHGF at all investigated time points was associated with shorter OS without any exception (after surgery: HR = 0.34, 95% CI: 0.14–0.85, *P* = 0.021; before chemotherapy: HR = 0.44, 95% CI: 0.22–0.89, *P* = 0.023; after the first three cycles of chemotherapy: HR = 0.41, 95% CI: 0.17–0.98, *P* = 0.045; and after the completion of chemotherapy: HR = 0.39, 95% CI: 0.19–0.80, *P* < 0.010; Fig. S6). Consistent results were obtained by the Kaplan–Meier analysis (Fig. 3B‐E).

Comparing other established risk factors of ovarian cancer, multivariate analysis revealed that a higher level of sHGF at each investigated time point was an independent predictor for either shorter PFS (after three cycles of chemotherapy: HR = 0.37, 95% CI: 0.17–0.20, *P* = 0.012), shorter OS (before chemotherapy: HR = 0.44, 95% CI: 0.44–0.94, *P* = 0.035), or both shorter PFS and OS (PFS after surgery: HR = 0.29, 95% CI: 0.14–0.63, *P* = 0.001; OS after surgery: HR = 0.09, 95% CI: 0.02–0.36, *P* < 0.001; PFS after chemotherapy: HR = 0.48, 95% CI: 0.25–0.92, *P* = 0.027; and OS after chemotherapy: HR = 0.43, 95% CI: 0.20–0.91, *P* = 0.019; Fig. S7).

### 3.5 A patient’s individual dynamic of sHGF is an independent predictor of survival

**Fig. 4:**
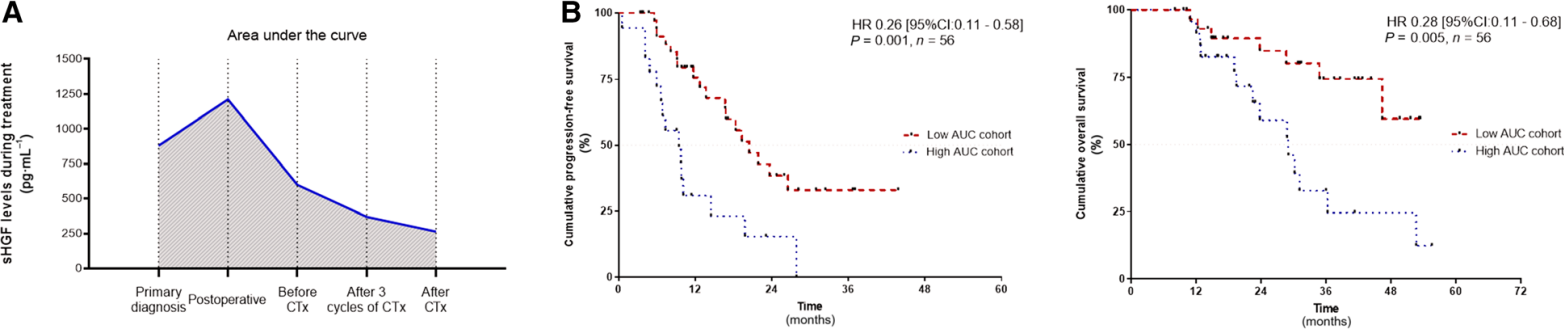
Prognostic relevance of the patients` individual sHGF dynamics. Patients’ dynamic curves showing the progression of sHGF levels in the time between primary diagnosis and after the completion of chemotherapy. (A) Example of a patient with a high AUC and (B) the Kaplan–Meier analyses comparing progression‐free survival and OS (*n*_(PFS and OS)_ = 56) of ovarian cancer patients with a high AUC value vs. patients with a low AUC value. *P*‐values, HR, and 95%CI were calculated, as described in Materials and methods section.

In the above‐mentioned analyses, prognostic relevance of sHGF was investigated independently at the different time points, without considering a patient’s individual course of sHGF over time. We investigated whether a patient’s individual progression of sHGF may also offer prognostic value, which would be a key component for sHGF‐guided therapy monitoring. This analysis was possible in a subset of 56 of 113 patients, from whom a set of five serum samples (primary diagnosis to the completion of chemotherapy) was available. Assuming the sHGF levels between the investigated samples were linear and nonfluctuating, we plotted a dynamic curve for each patient and calculated a corresponding AUC value. This reflected the individuals’ sHGF dynamic over the course of treatment (Fig. 4A), and patients were grouped into ‘AUC high’ or ‘AUC low’. The progression of sHGF was prognostically significant, and patients with a high AUC value had shorter PFS and OS, as indicated by the univariate analysis (PFS: HR = 0.34, 95% CI: 0.17–0.68, *P* = 0.002; OS: HR = 0.22, 95% CI: 0.09–0.55, *P* = 0.001) and the Kaplan–Meier analysis (PFS: HR = 0.26, 95% CI: 0.11–0.58, *P* = 0.001; and OS: HR = 0.28, 95% CI: 0.11–0.68, *P* = 0.005; Fig. 4B). Multivariate analysis confirmed that this was also independent from known risk factors (PFS: HR = 0.24, 95% CI: 0.11–0.53, *P* < 0.001; and OS: HR = 0.21, 95% CI: 0.07–0.63, *P* = 0.005).

### 3.6 sHGF is an independent prognostic biomarker in BRCA1/2 wild‐type ovarian cancer patients

In 57 of 113 patients, information on BRCA1/2 mutational status was available. Of these patients, 34 of 113 patients (30%) were BRCA1/2 wild‐type (wtBRCA1/2), whereas 23 of 113 patients (20.4%) had a pathogenic somatic or germline BRCA1/2 mutation. In the subgroup of wtBRCA1/2 patients, increased sHGF level at primary diagnosis was an independent predictor for shorter PFS (HR = 0.21, 95% CI: 0.06–0.78, *P* < 0.020) and OS (HR = 0.42, 95% CI: 0.03–0.91, *P* < 0.040; Fig. S8).

## 4 Discussion

This study investigated the clinical significance of sHGF as a serological biomarker for ovarian cancer. Importantly, we reported that a higher sHGF level is an independent predictor for survival at primary diagnosis and throughout primary treatment.

Circulating sHGF offers several advantages over traditional tissue‐based biomarkers. Cancerous tissue of the primary (chemotherapy‐naive) tumor is only histologically available at primary debulking surgery. Given the aim of surgical debulking is to achieve a macroscopic complete resection, it is not possible to obtain tissue throughout chemotherapy in a significant proportion of patients. Therefore, a clear advantage of a blood‐based biomarker is the availability of prognostic information at primary diagnosis and also throughout treatment by readily obtained blood samples. This is referred to as therapeutic monitoring. In addition, blood samples can be obtained alongside standard minor clinical procedures, not requiring surgery or specialist equipment at the point of sample collection. A tissue‐based biomarker could additionally be confounded by tumor heterogeneity, which is presumed to be less problematic in any blood‐based readout. This is due to the fact that analytes derived from different tumor (sub)clones and/or sites are ‘pooled’ in circulation, which may provide a more complete representation of the respective markers, if their serological concentrations are within the detection limits of the chosen method.

It was reported that HGF is detectable in primary ovarian cancer tissue and its level is increased in higher tumor stages [25, 29]. Moreover, HGF is present at high concentrations in malignant ascites of ovarian cancer patients and induces migration of human peritoneal mesothelial cells by activation of cMET [30]. A previous study reported elevated sHGF levels in ovarian cancer patients at primary diagnosis compared with patients with borderline tumors or benign disease [25]. In line with these findings, we report that sHGF at primary diagnosis is also highly elevated compared with healthy controls and allows reasonable discrimination between diseased and healthy individuals (AUC = 0.71). Future investigation with independently selected patient cohorts would be necessary, in order to assess the use of sHGF as a possible screening marker for ovarian cancer, as low‐stage ovarian cancer was underrepresented in the present unselected cohort (11.5% of patients; *n* = 13/113). Therefore, a statistically substantiated conclusion on the diagnostic capacity of sHGF for blood‐based detection of low‐stage ovarian cancer was beyond the scope of the present study.

Two possible scenarios could explain the underlying increased sHGF levels in ovarian cancer patients. Firstly, HGF‐dependent cMET activation in ovarian tumors could be exerted by an autocrine loop [31], by which HGF is expressed and excreted by the tumor cells themselves. In this scenario, the cancerous tissue itself may contribute to an elevated sHGF level in blood, which is supported by previous studies showing that (a) HGF is present in malignant ascites [32] and that (b) HGF expression in ovarian cancer tissue parallels (increasing) tumor stage [29]. Secondly, considering circulating sHGF throughout the body [33], cMET activation of tumor cells could also be regulated by paracrine mechanisms. Therefore, cancer cells would be dependent on HGF‐producing sources, such as neighboring tumor‐associated fibroblasts [34] or distant endogenous sources. However, determining the precise origin of sHGF in ovarian cancer patients is complex because elevated HGF levels are not unequivocally specific for cancer, as they were also reported in infections or graft‐versus‐host disease [35, 36].

Nevertheless, our data suggest that sHGF level reflects advanced disease because (a) it was associated with an increased FIGO stage and (b) declined rapidly after initiating chemotherapy. In addition, we observed a strong yet transient sHGF surge after primary surgery. It seems likely that physical traumata, conferred by surgery, may alter the rate of HGF release into circulation at this point of treatment. This is consistent with a study showing that HGF levels transiently increase following thoracic surgery [37]. Increased HGF and cMET levels were also observed after, for example, heart injury, suggesting that the HGF/cMET pathway is involved in tissue damage response and tissue regeneration [38, 39, 40].

A preliminary study showed that patients with advanced ovarian cancer and increased preoperative sHGF have a shorter disease‐free survival [25]. To the best of our knowledge, our present study is the first to advance these findings with a systematic investigation of longitudinal sHGF levels during primary treatment and at disease relapse, also potentially allowing disease and/or therapeutic monitoring. Our study shows that a high sHGF level is an independent predictor of poor outcome at all stages of primary treatment. Therefore, sHGF detection may be suitable for identifying patients with a more aggressive disease, that is, those with a high risk of recurrence and higher mortality. Our study offers new insights into the potential clinical use of sHGF as a discrete blood‐based biomarker, which may be used in combination with CA125 and could repeatedly be applied for prognostic stratification, for example, in terms of sHGF‐guided therapy monitoring. In addition, the potential of longitudinal sHGF levels as a dynamic biomarker for predicting/monitoring response to HGF/cMET targeting drugs could be explored in future [23, 24, 41].

In the present study, sHGF was not predictive for platinum resistance, possibly due to the limited number of patients with platinum resistance in our cohort (*n* = 12/113). Therefore, we encourage future analysis in a larger set of patients with platinum‐resistant ovarian cancer, in order to study the predictive value of sHGF. It is important to note that the clinical definition of platinum resistance may likely change due to recent new therapeutic options in maintenance therapy after (partial/complete) response to first‐line platinum‐based chemotherapy. This includes the approval of maintenance therapy with the PARP inhibitor niraparib independent of the biomarker status [6], or the combined treatment with the PARP inhibitors olaparib and bevacizumab in BRCA1/2‐mutant or HRD‐positive ovarian cancer [9]. Since the greatest benefit of these novel treatment regimens is most strikingly seen in patients with BRCA1/2‐mutant or HRD‐positive ovarian cancer, and less pronounced in wtBRCA1/2 and HRD‐negative ovarian cancers, there is a clinical need to identify high‐risk patients with wtBRCA1/2/HRD‐negative ovarian cancers. This could potentially be achieved by sHGF‐guided risk stratification.

Since circulating tumor cells (CTCs) are a central element of recent liquid biopsy studies on ovarian cancer [42, 43, 44], our data encourage further investigation on the relationship between sHGF levels and CTCs. Considering the already confirmed protumorigenic role of the HGF/cMET pathway in ovarian cancer [22], there might be a biologically relevant interaction between HGF/cMET signaling and micrometastasis dynamics. If an association can be identified, this could in turn be modulated by HGF/cMET‐directed therapies. Moreover, a combined analysis of HGF levels and CTC counts may further improve prognostic relevance.

## 5 Conclusion

This is the first study proposing sHGF as a blood‐based and independent prognostic biomarker for ovarian cancer patients at primary diagnosis and in the course of primary treatment. In addition to the CA125 tumor marker, the detection of sHGF could readily be implemented into routine clinical diagnostics to support individualized prognostic stratification and sHGF‐guided therapy monitoring in ovarian cancer. Patients with high risk of recurrence, as identified by an increased sHGF level, might benefit from intensified therapy regimes, such as additional immunotherapy.

## Conflict of interest

DMK has a patent application pending regarding the use of HGF as a prognostic biomarker in ovarian cancer. With regard to the present study, all other authors have declared that no further conflict of interest exists.

## Data Accessibility

All relevant data and descriptions of statistical workflows are contained in the manuscript. Raw data of ELISA‐based HGF detection can be made available from the first author (Daniel.m.klotz@ukdd.de) upon reasonable request.

## Author contributions

DMK, JDK, PW, and TL made substantial contributions to the conception and design of the study. DMK and JDK contributed to the experimental work or to the acquisition of data and to the analysis/interpretation of the results. JDK and DMK were involved in drafting the manuscript, creating figures, and revising the manuscript. DMK initiated the study. All authors read and approved the manuscript in its final version.

## Supporting information


**Fig. S1.** Graphical and numerical summary of sHGF cutoff determination. (A) Determination of fixed sHGF level cutoffs, categorizing patients into sHGF‐high and sHGF‐low group by maximally selected rank statistics, with graphical example shown for sHGF at primary diagnosis (OS). (B) List of fixed cutoffs and the calculated p‐values for the indicated univariate, multivariate Cox proportional hazard regression model and Kaplan‐Meier analyses. Ovarian cancer patients primary diagnosis *n*_(PFS)_ = 96 and *n*_(OS)_ = 100, after primary surgery *n*_(OS and PFS)_ = 56, before chemotherapy *n*_(PFS)_ = 73 and *n*_(OS)_ = 74, after three cycles of chemotherapy *n*_(PFS and OS)_ = 56, after chemotherapy *n*_(PFS and OS)_ = 75, AUC cohort *n*_(PFS and OS)_ = 56 and wtBRCA cohort *n*_(PFS and OS)_ = 34. Cutoffs were determined by maximally selected rank statistics.

**Fig. S2.** A Diagnostic capacity of sHGF level at primary diagnosis and Supplementary Figure 2 B sHGF according to histological subtype at primary diagnosis. (A) Receiver operating characteristic (ROC) analysis to determine the diagnostic ability of sHGF level to distinguish between ovarian cancer patients (FIGO I and II, *n* = 13) and healthy controls (*n* = 82). The respective area under the curve (AUC) value and the 95% confidence interval (CI) are indicated. (B) Scatter plots comparing sHGF level between nonserous and serous histologic subtypes (*n* = 100). The black horizontal lines indicate the median sHGF levels in each group with error bars, showing the interquartile range. *P*‐value, according to the nonparametric, two‐tailed Mann–Whitney *U*‐test, is indicated.

**Fig. S3.** Scatter plots comparing sHGF level between patients with and without residual tumor left after primary debulking at primary surgery or within one week after surgery. Scatter plots comparing sHGF level between patients with and without residual tumor left after primary debulking at primary diagnosis (*n* = 100) or within one week after surgery (postoperative, *n* = 56). The black horizontal lines indicate the median sHGF levels in each group with error bars, showing the interquartile range. *P*‐values, according to the nonparametric, two‐sided Mann–Whitney test, are indicated.

**Fig. S4.** sHGF and relapsed disease. (A) Spearman correlation analysis of sHGF levels at primary diagnosis and sHGF at relapse with linear regression (red line) shown, matched samples available (*n* = 15) (B) Kaplan‐Meier analysis comparing overall survival (OS) of patients with high sHGF level *vs*. patients with low sHGF level after relapse (*n* = 28). Cutoff was the median with 796.8 ng·mL^‐1^ and *P*‐values, hazard ratio (HR) and 95% confidence intervals (95%CI) were calculated, as described in Methods section.

**Fig. S5.** Predicting platinum resistance by measuring sHGF throughout primary treatment. Results from univariate and multivariate generalized linear model analyses at all investigated time points, including odds ratio (OR) and 95% CIs and *P*‐values, with *P* < 0.05 indicates statistical, as described in the methods section. Ovarian cancer patients: *n*_(primary diagnosis)_ = 96, *n*_(after surgery)_ = 56, *n*_(before chemotherapy)_ = 73, *n*_(after three cycles of chemotherapy)_ = 56 and *n*_(after chemotherapy)_ = 75. Distinct cutoffs have been used as shown and described in the Methods section.

**Fig. S6.** Univariate analysis and prognostic relevance of sHGF level. (A) Results from univariate Cox proportional hazard regression model analyses at all investigated time points, including hazard ratio (HR) and 95% CIs and *P*‐values, with *P* < 0.05 indicates statistical significance, as described in the methods section. Ovarian cancer patient at primary diagnosis *n*_(PFS)_ = 96 and *n*_(OS)_ = 100, after primary surgery *n*_(OS and PFS)_ = 56, before chemotherapy *n*_(PFS)_ = 73 and *n*_(OS)_ = 74, after three cycles of chemotherapy *n*_(PFS and OS)_ = 56, after chemotherapy *n*_(PFS and OS)_ = 75, AUC cohort *n*_(PFS and OS)_ = 56. (B) Graphical analysis of HRs with regard to progression‐free survival (PFS) and overall survival (OS).

**Fig. S7.** Multivariate analysis and prognostic relevance of sHGF level. (A) Results from multivariate Cox proportional hazard regression model analyses at all investigated time points, including hazard ratio (HR) and 95% CIs and *P*‐values, with *P* < 0.05 indicates statistical significance, as described in the methods section. Ovarian cancer patient at primary diagnosis *n*_(PFS)_ = 96 and *n*_(OS)_ = 100, after primary surgery *n*_(OS and PFS)_ = 56, before chemotherapy *n*_(PFS)_ = 73 and *n*_(OS)_ = 74, after three cycles of chemotherapy *n*_(PFS and OS)_ = 56, after chemotherapy *n*_(PFS and OS)_ = 75, AUC cohort *n*_(PFS and OS)_ = 56. (B) Graphical analysis of HRs with regard to progression‐free survival (PFS) and overall survival (OS).

**Fig. S8.** Univariate and multivariate analysis of sHGF level in wtBRCA1/2 cohort at primary diagnosis. Results from univariate and multivariate Cox proportional hazard regression model analyses from the subcohort of patients with wtBRCA1/2 ovarian cancer at primary diagnosis (*n* = 34), including hazard ratio (HR) and 95% CIs and *P*‐values, with *P* < 0.05 indicates statistical significance, as described in the methods section.
