# Supplementary figures and images for: Prognostic relevance of longitudinal HGF levels in serum of patients with ovarian cancer
Source: Mol Oncol. 2021 Apr 2;15(12):3626–38. doi: 10.1002/1878-0261.12949 (PMC8637578; doi:10.1002/1878-0261.12949)

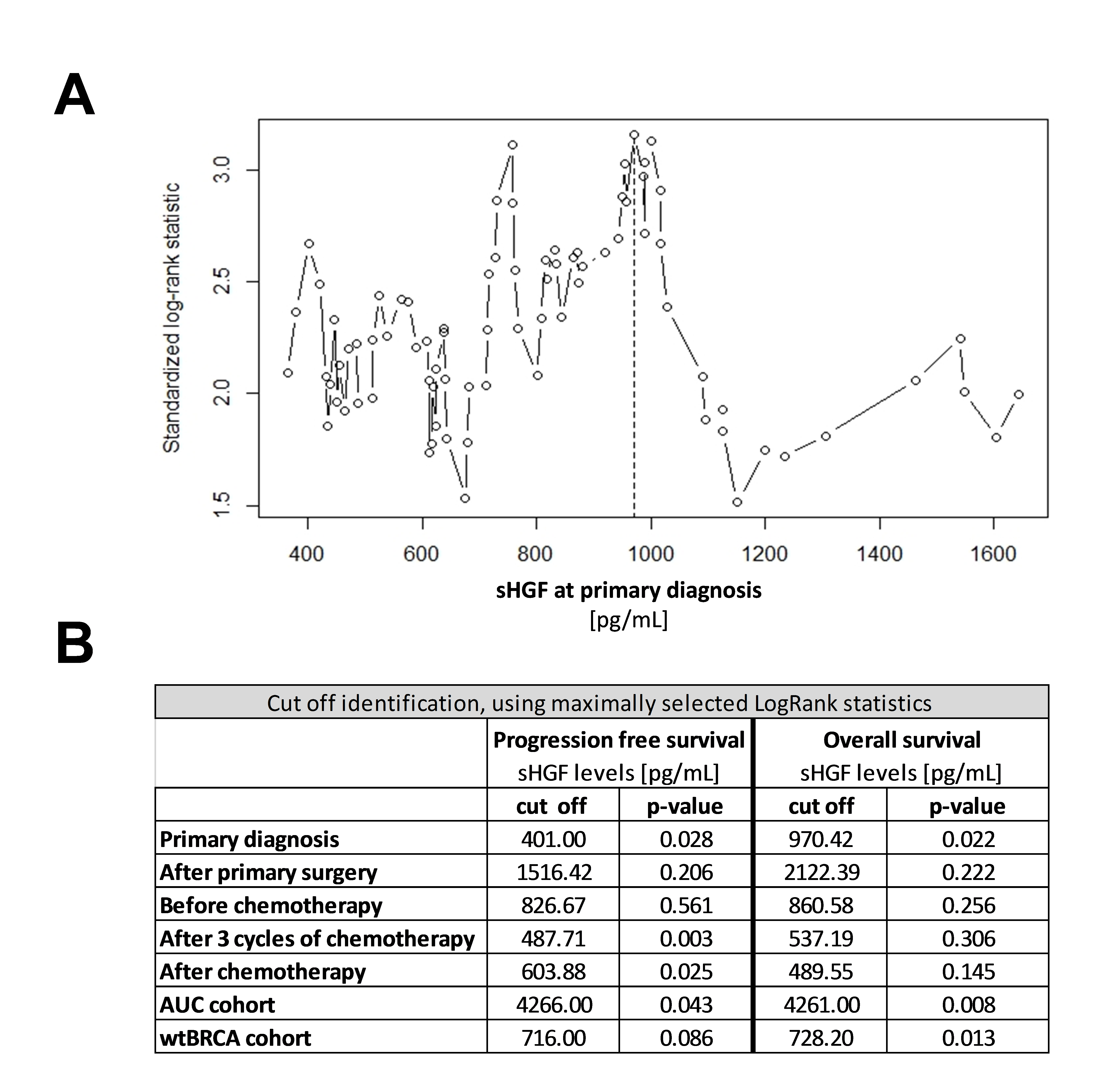

Supplement: Supplementary file 1 — Fig. S1. Graphical and numerical summary of sHGF cutoff determination. (A) Determination of fixed sHGF level cutoffs, categorizing patients into sHGF‐high and sHGF‐low group by maximally selected rank statistics, with graphical example shown for sHGF at primary diagnosis (OS). (B) List of fixed cutoffs and the calculated p‐values for the indicated univariate, multivariate Cox proportional hazard regression model and Kaplan‐Meier analyses. Ovarian cancer patients primary diagnosis n (PFS) = 96 and n (OS) = 100, after primary surgery n (OS and PFS) = 56, before chemotherapy n (PFS) = 73 and n (OS) = 74, after three cycles of chemotherapy n (PFS and OS) = 56, after chemotherapy n (PFS and OS) = 75, AUC cohort n (PFS and OS) = 56 and wtBRCA cohort n (PFS and OS) = 34. Cutoffs were determined by maximally selected rank statistics. [file MOL2-15-3626-s008.tif]

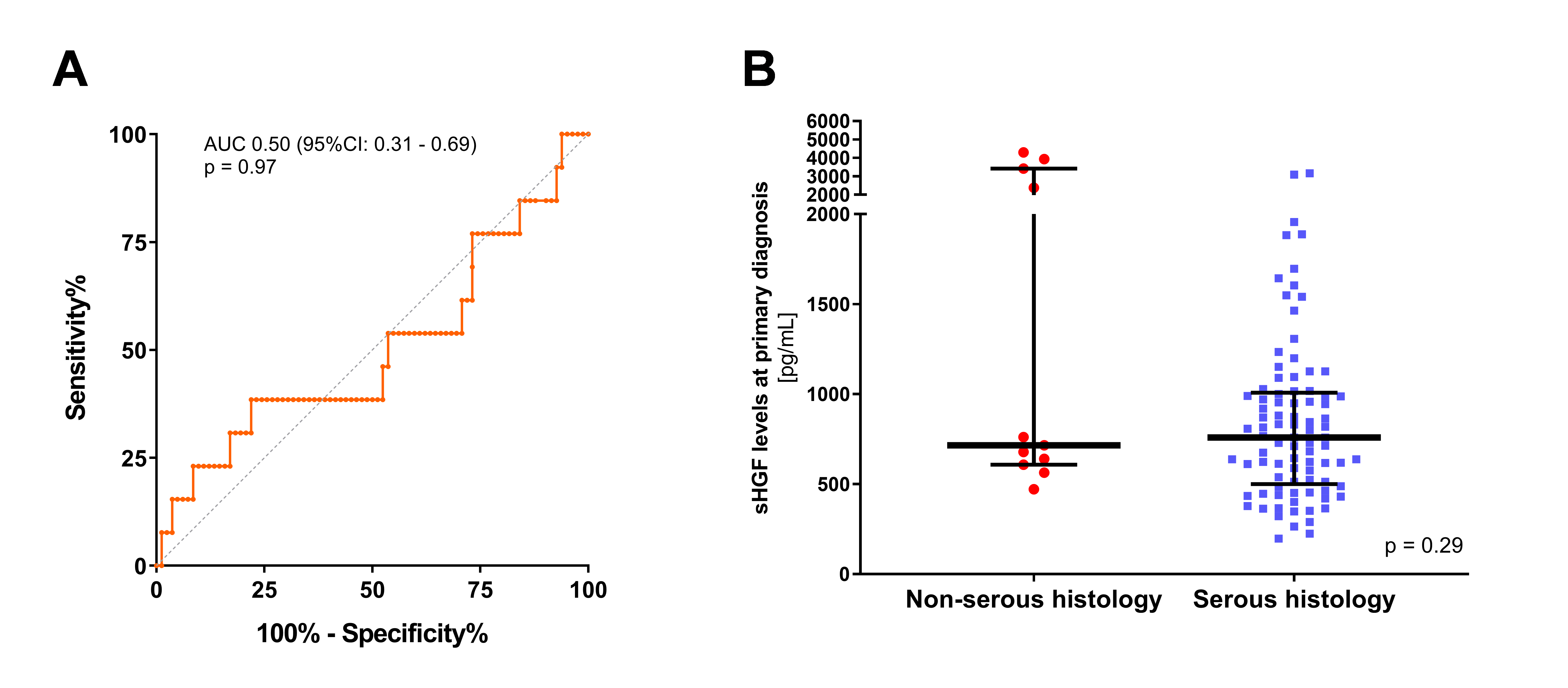

Supplement: Supplementary file 2 — Fig. S2. A Diagnostic capacity of sHGF level at primary diagnosis and Supplementary Figure 2 B sHGF according to histological subtype at primary diagnosis. (A) Receiver operating characteristic (ROC) analysis to determine the diagnostic ability of sHGF level to distinguish between ovarian cancer patients (FIGO I and II, n = 13) and healthy controls (n = 82). The respective area under the curve (AUC) value and the 95% confidence interval (CI) are indicated. (B) Scatter plots comparing sHGF level between nonserous and serous histologic subtypes (n = 100). The black horizontal lines indicate the median sHGF levels in each group with error bars, showing the interquartile range. P‐value, according to the nonparametric, two‐tailed Mann–Whitney U‐test, is indicated. [file MOL2-15-3626-s006.tif]

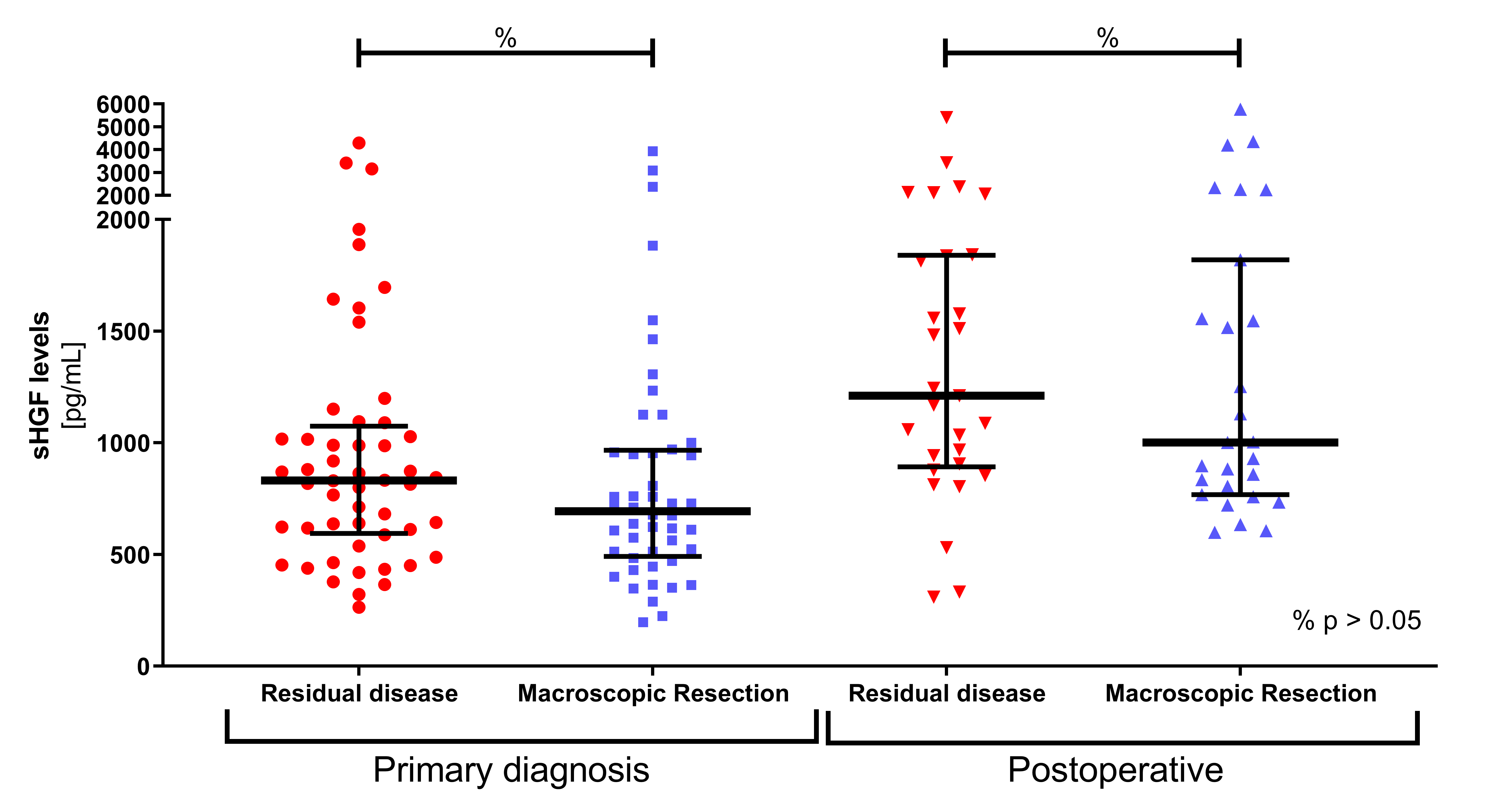

Supplement: Supplementary file 3 — Fig. S3. Scatter plots comparing sHGF level between patients with and without residual tumor left after primary debulking at primary surgery or within one week after surgery. Scatter plots comparing sHGF level between patients with and without residual tumor left after primary debulking at primary diagnosis (n = 100) or within one week after surgery (postoperative, n = 56). The black horizontal lines indicate the median sHGF levels in each group with error bars, showing the interquartile range. P‐values, according to the nonparametric, two‐sided Mann–Whitney test, are indicated. [file MOL2-15-3626-s005.tif]

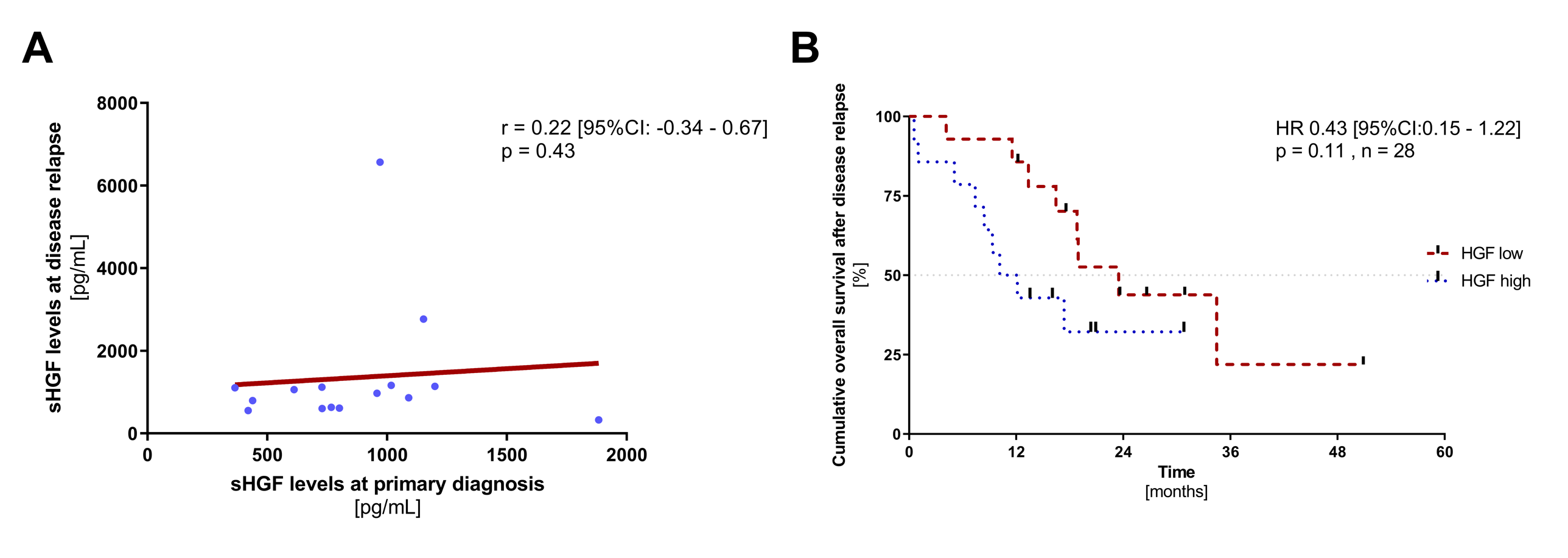

Supplement: Supplementary file 4 — Fig. S4. sHGF and relapsed disease. (A) Spearman correlation analysis of sHGF levels at primary diagnosis and sHGF at relapse with linear regression (red line) shown, matched samples available (n = 15) (B) Kaplan‐Meier analysis comparing overall survival (OS) of patients with high sHGF level vs. patients with low sHGF level after relapse (n = 28). Cutoff was the median with 796.8 ng·mL‐1 and P‐values, hazard ratio (HR) and 95% confidence intervals (95%CI) were calculated, as described in Methods section. [file MOL2-15-3626-s007.tif]

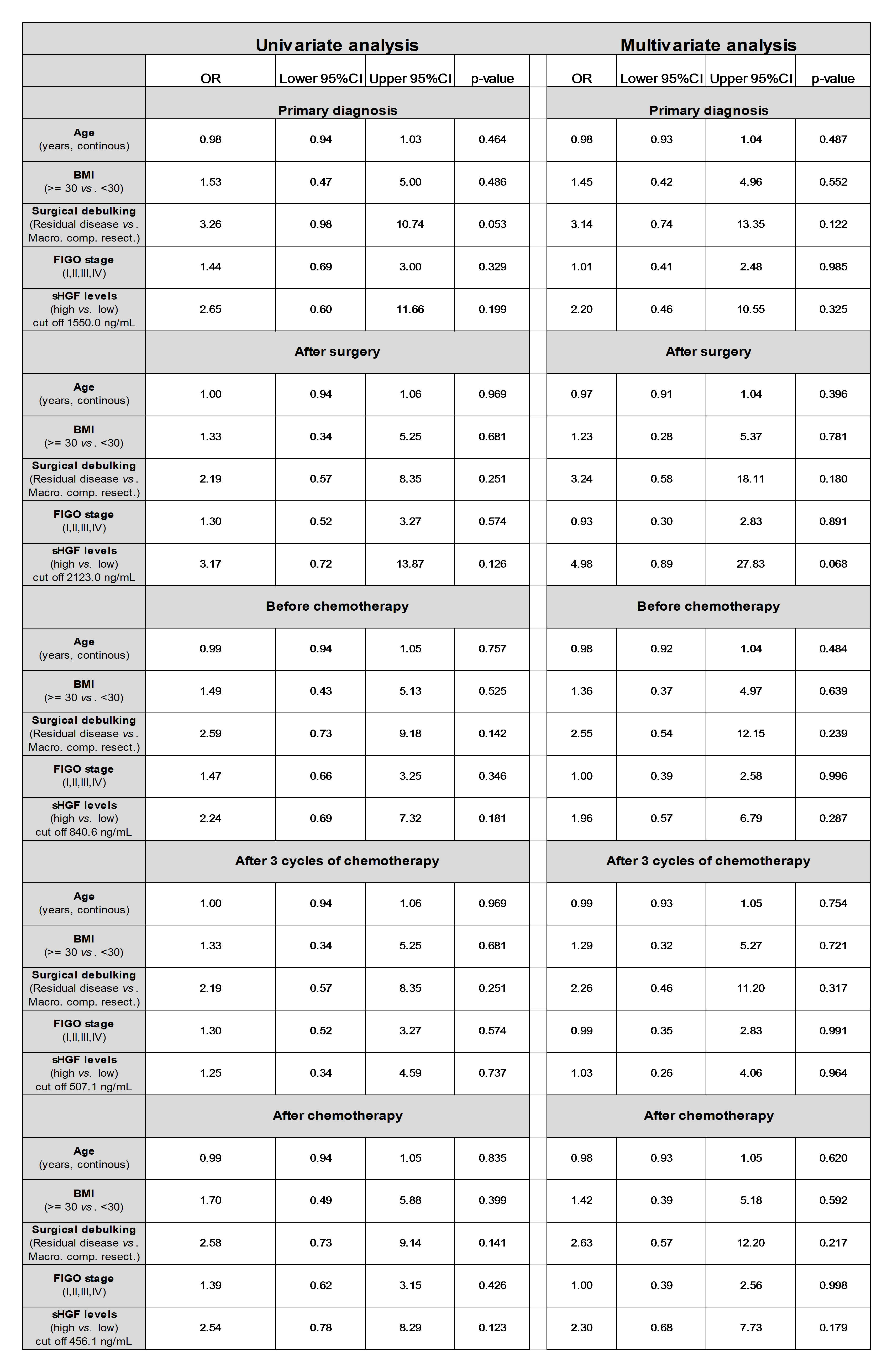

Supplement: Supplementary file 5 — Fig. S5. Predicting platinum resistance by measuring sHGF throughout primary treatment. Results from univariate and multivariate generalized linear model analyses at all investigated time points, including odds ratio (OR) and 95% CIs and P‐values, with P < 0.05 indicates statistical, as described in the methods section. Ovarian cancer patients: n (primary diagnosis) = 96, n (after surgery) = 56, n (before chemotherapy) = 73, n (after three cycles of chemotherapy) = 56 and n (after chemotherapy) = 75. Distinct cutoffs have been used as shown and described in the Methods section. [file MOL2-15-3626-s004.tif]

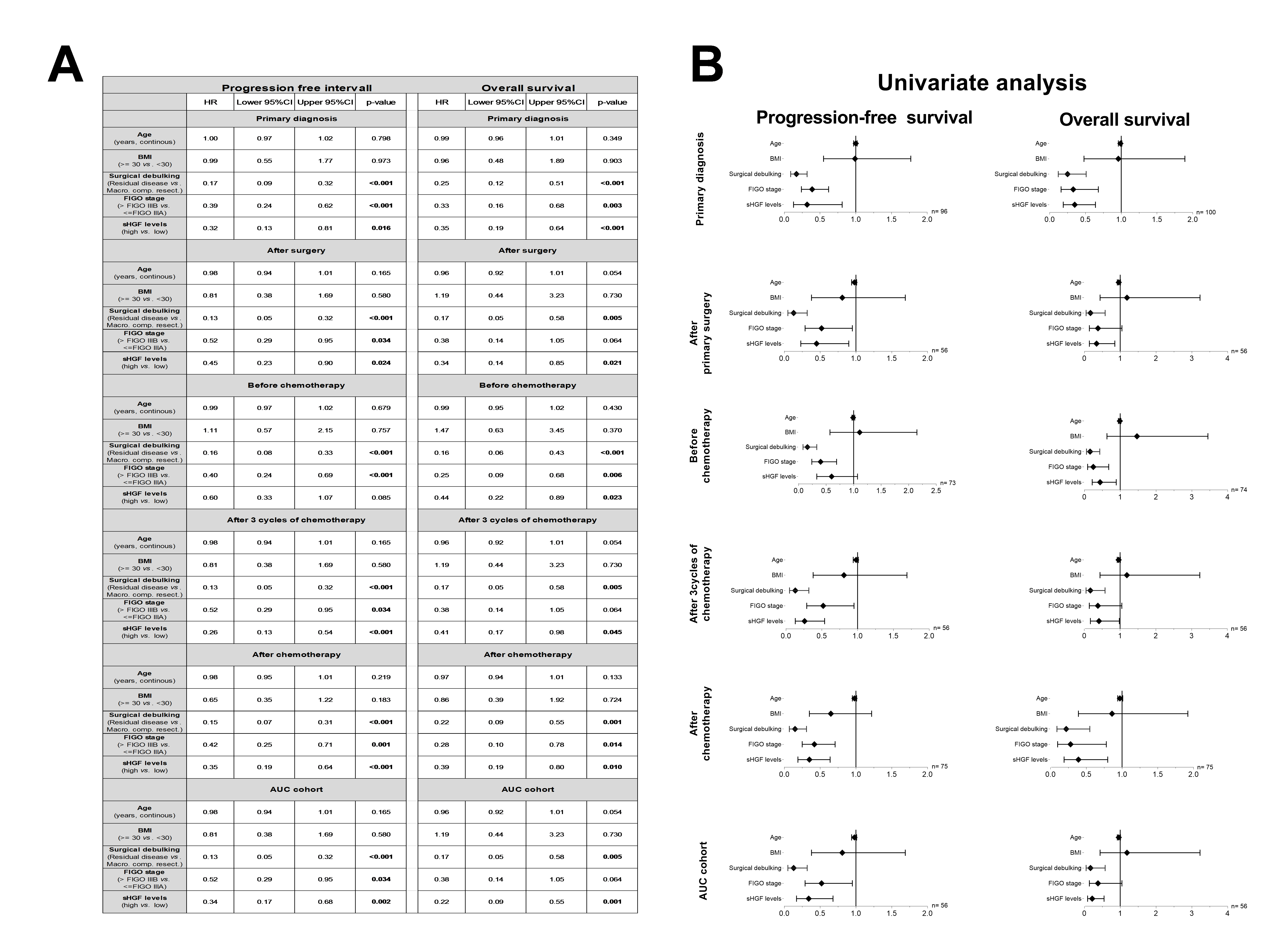

Supplement: Supplementary file 6 — Fig. S6. Univariate analysis and prognostic relevance of sHGF level. (A) Results from univariate Cox proportional hazard regression model analyses at all investigated time points, including hazard ratio (HR) and 95% CIs and P‐values, with P < 0.05 indicates statistical significance, as described in the methods section. Ovarian cancer patient at primary diagnosis n (PFS) = 96 and n (OS) = 100, after primary surgery n (OS and PFS) = 56, before chemotherapy n (PFS) = 73 and n (OS) = 74, after three cycles of chemotherapy n (PFS and OS) = 56, after chemotherapy n (PFS and OS) = 75, AUC cohort n (PFS and OS) = 56. (B) Graphical analysis of HRs with regard to progression‐free survival (PFS) and overall survival (OS). [file MOL2-15-3626-s001.tif]

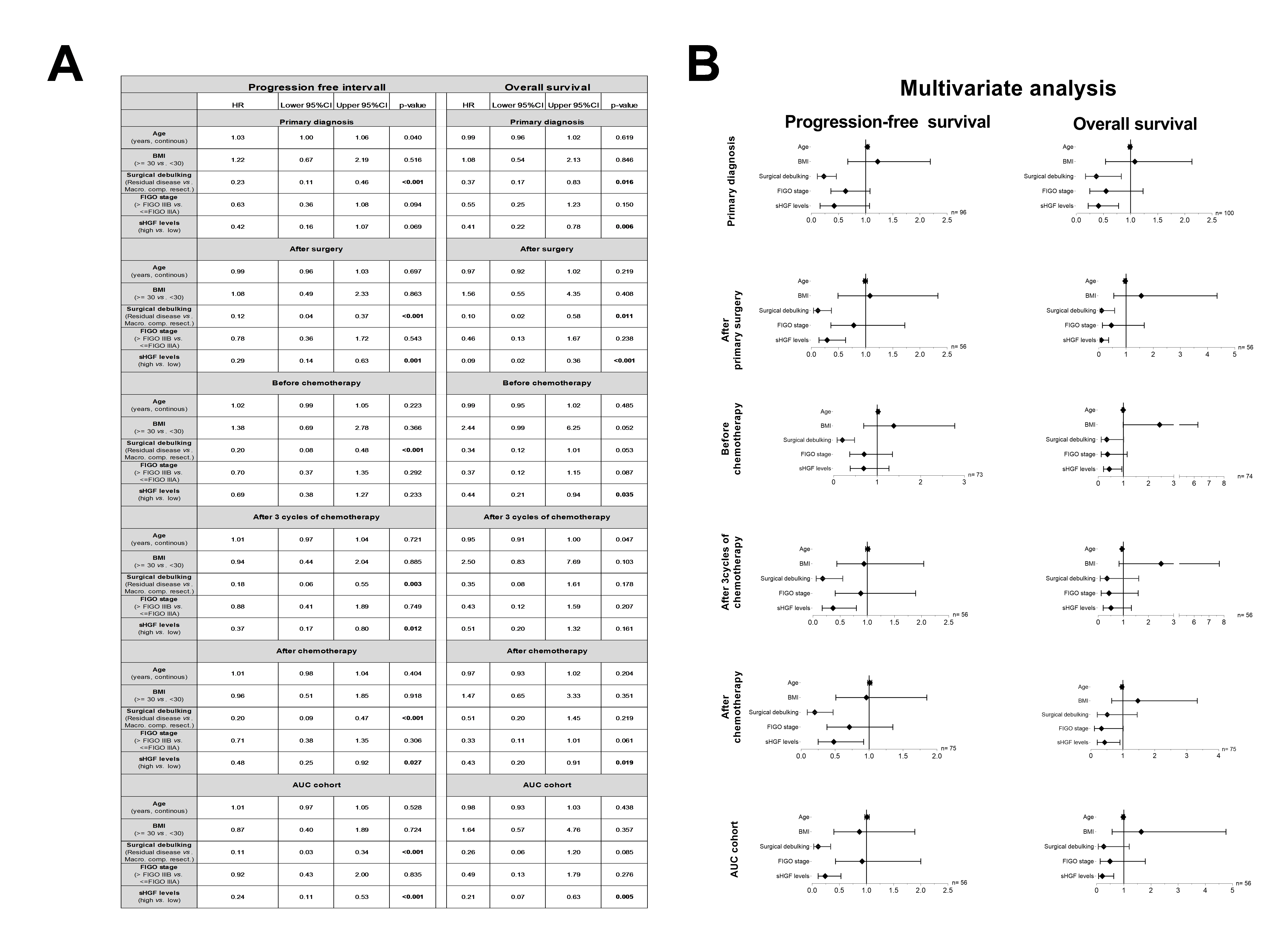

Supplement: Supplementary file 7 — Fig. S7. Multivariate analysis and prognostic relevance of sHGF level. (A) Results from multivariate Cox proportional hazard regression model analyses at all investigated time points, including hazard ratio (HR) and 95% CIs and P‐values, with P < 0.05 indicates statistical significance, as described in the methods section. Ovarian cancer patient at primary diagnosis n (PFS) = 96 and n (OS) = 100, after primary surgery n (OS and PFS) = 56, before chemotherapy n (PFS) = 73 and n (OS) = 74, after three cycles of chemotherapy n (PFS and OS) = 56, after chemotherapy n (PFS and OS) = 75, AUC cohort n (PFS and OS) = 56. (B) Graphical analysis of HRs with regard to progression‐free survival (PFS) and overall survival (OS). [file MOL2-15-3626-s002.tif]

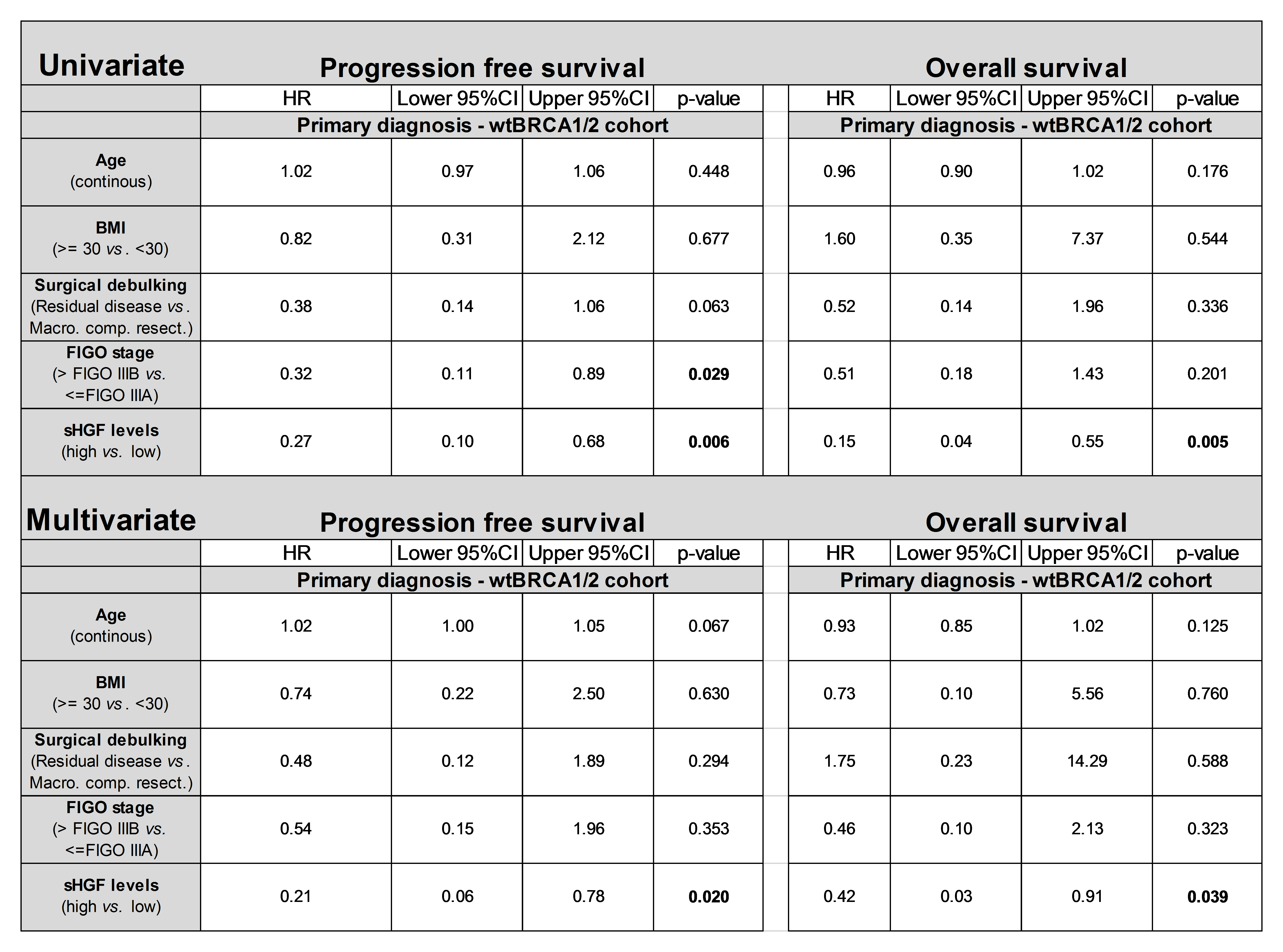

Supplement: Supplementary file 8 — Fig. S8. Univariate and multivariate analysis of sHGF level in wtBRCA1/2 cohort at primary diagnosis. Results from univariate and multivariate Cox proportional hazard regression model analyses from the subcohort of patients with wtBRCA1/2 ovarian cancer at primary diagnosis (n = 34), including hazard ratio (HR) and 95% CIs and P‐values, with P < 0.05 indicates statistical significance, as described in the methods section. [file MOL2-15-3626-s003.tif]
